# First person – Patricia Shaw

**DOI:** 10.1242/dmm.047076

**Published:** 2020-09-25

## Abstract

First Person is a series of interviews with the first authors of a selection of papers published in Disease Models & Mechanisms, helping early-career researchers promote themselves alongside their papers. Patricia Shaw is first author on ‘Longitudinal neuroanatomical and behavioral analyses show phenotypic drift and variability in the Ts65Dn mouse model of Down syndrome’, published in DMM. Patricia is a PhD student in the lab of Tarik Haydar at Boston University School of Medicine, USA, researching the underlying genetic and cellular mechanisms that contribute to brain development, and investigating how these processes are altered in diseases and disorders, such as Down syndrome, in order to identify novel targetable approaches for therapeutics.


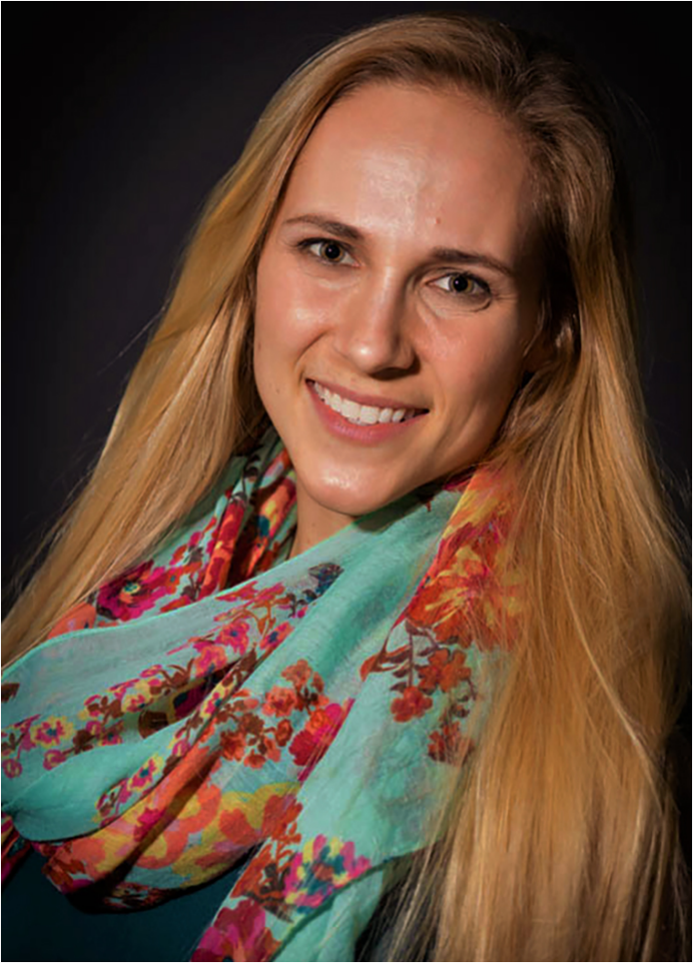


**Patricia Shaw**

**How would you explain the main findings of your paper to non-scientific family and friends?**

In order to fully understand how the brain is changed in Down syndrome, we rely on mouse models that allow us to look more closely at the genetic, molecular, cellular and functional components that contribute to intellectual disability in people with Down syndrome. However, in order for these models to be useful, they must display disease-relevant characteristics that resemble what we see in people with Down syndrome. Our findings show that the most commonly used mouse model, Ts65Dn, does not reliably display Down syndrome-like alterations in neurodevelopment and behaviour, highlighting a major limitation of its use. We investigated how brain development, structure and function are changed during gestation, as well as throughout adulthood in Ts65Dn mice, and found that, depending on which generation of Ts65Dn is studied, results are subject to variability. We detail factors that could be contributing to this, including genetic diversity due to the background strain, as well as selective breeding practices. Additionally, we outline steps researchers should take to address the problem of variability within their own studies. Our work here represents the first longitudinal cohort-based comparison of Ts65Dn across time and challenges the use of Ts65Dn as an effective model for studying Down syndrome. This is important as there are currently no therapeutics available for people with Down syndrome to help curtail the cognitive deficits. These lack of treatments could be due to the widespread use of Ts65Dn in developing and testing therapies.

“Our findings show that the most commonly used mouse model, Ts65Dn, does not reliably display Down syndrome-like alterations in neurodevelopment and behaviour, highlighting a major limitation of its use.”

**What are the potential implications of these results for your field of research?**

The Ts65Dn mouse model has been a cornerstone of Down syndrome research since its development in 1990, and discoveries made using this model helped to expand our understanding of the neuropathophysiology of Down syndrome. However, our findings suggest that the Ts65Dn model is subject to variability and phenotypic drift that may limit the use of this model for studying Down syndrome. The various drawbacks of the Ts65Dn model have been known for quite some time but have never been experimentally assessed or discussed before now. This study confronts the long-standing beliefs and assumptions in the field, and encourages other researchers to critically evaluate the future use of Ts65Dn for studying Down syndrome. Although our research has looked at the Ts65Dn model specifically, the problems we have identified could be present in other mouse models of disease as well. We hope our findings presented here highlight the need for the robust and rigorous assessment of animal models if they are to be responsibly used for translational research.

**What are the main advantages and drawbacks of the model system you have used as it relates to the disease you are investigating?**

The use of mouse models to study the detailed complexities of human diseases is necessary to advance the development of treatments. Mouse models provide a level of experimental manipulation that is necessary to uncover many of the fundamental components of disease that cannot be achieved with human studies alone. However, our research emphasizes the major drawbacks of using Ts65Dn mice, which include the genetic makeup and breeding of the mice that might contribute to the phenotypic variability and drift within the line. We hope that with advances in engineering technology, new mouse models can be generated that more closely resemble the genetic and phenotypic makeup of Down syndrome, and can be used in parallel with other model systems, such as patient-derived induced pluripotent stem cells, to elucidate the underlying mechanisms of Down syndrome.

**Figure:**
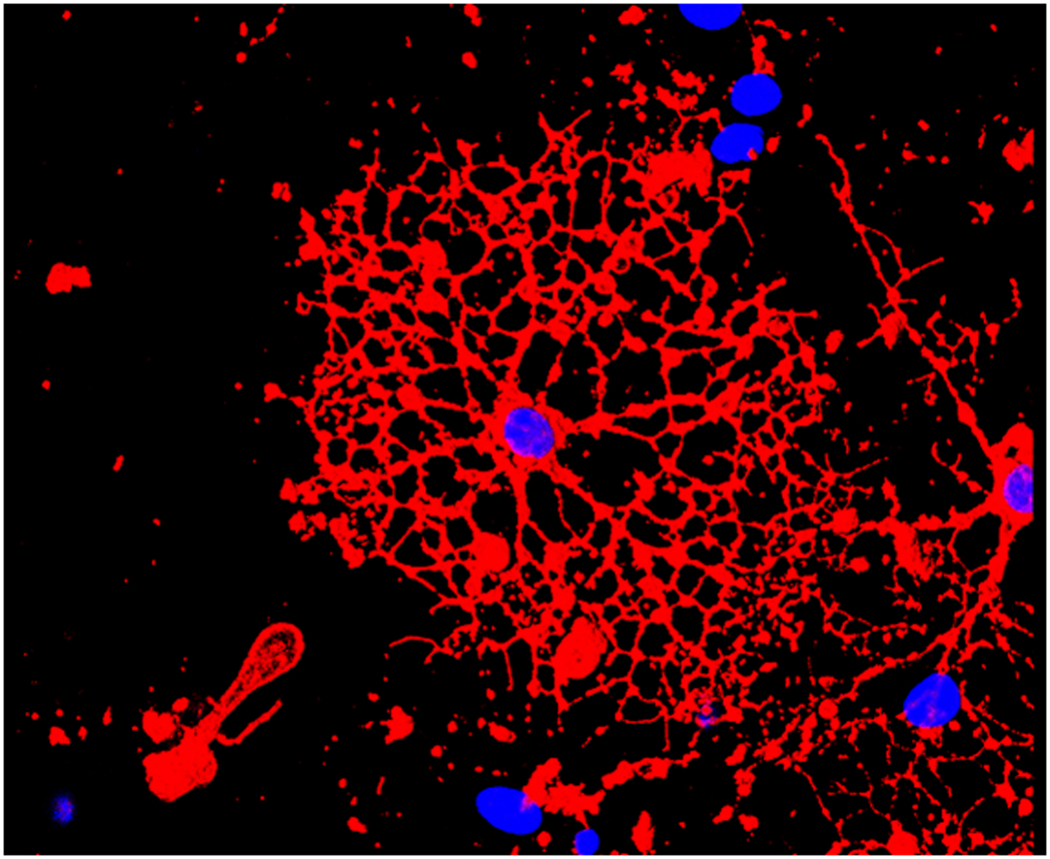
**Maturing oligodendrocyte cultured *in vitro* from Ts65Dn mouse brains stained for RmAb.**

**Describe what you think is the most significant challenge impacting your research at this time and how will this be addressed over the next 10 years?**

Our understanding of the underlying mechanisms that contribute to the hallmark features of Down syndrome has greatly increased over the years. However, there are still no options available for people with Down syndrome in terms of treatments and therapies to improve their quality of life. Changing this will require more reliable and robust platforms with which we can study Down syndrome, greater collaboration between researchers in academia and industry, and increased transparency that will allow quality research to move forward. The number of clinical trials for Down syndrome, specifically the neurological consequences, is very low and we hope that in the coming years, with innovative advances in model development and partnerships with the pharmaceutical industry, we will be able to identify targetable approaches to treating Down syndrome that will provide individuals with a great quality of life.

**What's next for you?**

I enjoy studying neuroscience and the challenge that comes with trying to understand the beautiful intricacies of the brain. I look forward to continuing in this line of research and contributing to work that will benefit people who are affected by neurological diseases and disorders.
